# What are clinically relevant performance metrics in robotic surgery? A systematic review of the literature

**DOI:** 10.1007/s11701-022-01457-y

**Published:** 2022-10-03

**Authors:** Melissa M. Younes, Kirsten Larkins, Gloria To, Grace Burke, Alexander Heriot, Satish Warrier, Helen Mohan

**Affiliations:** 1 The University of Melbourne, 305 Grattan Street, Parkville, VIC Australia; 2 Peter MacCallum Cancer Centre, Melbourne, VIC Australia; 3 International Medical Robotics Academy, North Melbourne, VIC Australia; 4 Monash University, Clayton, VIC Australia; 5 Austin Health, Heidelberg, VIC Australia

**Keywords:** Clinically relevant performance metrics, Clinically relevant outcome measures, Proficiency-based training, Robotic surgical education

## Abstract

A crucial element of any surgical training program is the ability to provide procedure-specific, objective, and reliable measures of performance. During robotic surgery, objective clinically relevant performance metrics (CRPMs) can provide tailored contextual feedback and correlate with clinical outcomes. This review aims to define CRPMs, assess their validity in robotic surgical training and compare CRPMs to existing measures of robotic performance. A systematic search of Medline and Embase databases was conducted in May 2022 following the PRISMA guidelines. The search terms included Clinically Relevant Performance Metrics (CRPMs) OR Clinically Relevant Outcome Measures (CROMs) AND robotic surgery. The study settings, speciality, operative context, study design, metric details, and validation status were extracted and analysed. The initial search yielded 116 citations, of which 6 were included. Citation searching identified 3 additional studies, resulting in 9 studies included in this review. Metrics were defined as CRPMs, CROMs, proficiency-based performance metrics and reference-procedure metrics which were developed using a modified Delphi methodology. All metrics underwent both contents and construct validation. Two studies found a strong correlation with GEARS but none correlated their metrics with patient outcome data. CRPMs are a validated and objective approach for assessing trainee proficiency. Evaluating CRPMs with other robotic-assessment tools will facilitate a multimodal metric evaluation approach to robotic surgery training. Further studies should assess the correlation with clinical outcomes. This review highlights there is significant scope for the development and validation of CRPMs to establish proficiency-based progression curricula that can be translated from a simulation setting into clinical practice.

## Introduction

The need for high-quality robotic surgical training is becoming more relevant with the increasing uptake of robotic surgery across multiple specialities. A crucial element of any surgical training program is the ability to provide procedure-specific, objective, and reliable measures of performance [1]. Metric-based assessment in surgical training has been shown to improve trainee performance [1]. Proficiency-based training is a concept where trainees are given objective goals or benchmarks they are required to achieve at each level of surgical training, before progressing to the next [2]. It focuses on improving performance and maintaining the proficiency of that performance rather than relying on caseload as a representation of surgical skill [2]. It has been shown that this approach produces overall higher proficiency scores and reduced intra-operative complications in comparison to conventional operating-room training [3]. Hence, proficiency-based progression (PBP) training utilises simulation to allow trainees to achieve proficiency in a “risk-free environment” before operating on a patient and improve clinical outcomes [2]. However, to evaluate whether benchmarks have been achieved and provide feedback to trainees, surgical trainers require metrics to objectively assess performance [2]. Therefore, to meet the requirements of PBP training in robotic surgery, there is a need for validated metrics to provide tailored feedback and guide trainee progression.

Currently, automated performance metrics (APMs) are objective, reproducible measures derived from kinemetric data that assess surgical skill [4]. However, they are not readily available in live operating settings and thus lack translation from simulation to clinical contexts. Additionally, APMs rely on the availability of annotated datasets used to evaluate performance and the transferability of these datasets across various operating techniques, toolsets and procedures remain poor [5]. Similarly, several tools have been created and utilised to measure surgical proficiency during robotic surgery such as the Global Evaluative Assessment of Robotic Skills (GEARS). GEARS, though previously validated, provides overall proficiency feedback about robotic surgical skills by grading six domains without adapting them to be procedure specific [6–8]. It also remains reliant on assessor subjectivity and human rating which introduces the risk of bias [4]. Another tool, the Robotic Anastomosis Competency Evaluation (RACE), is a validated, objective scoring system to assess surgical performance during ureterovesical anastomosis (UVA) and provide structured feedback [9]. Whilst UVA is a critical step in surgical procedures, such as robot-assisted radical prostatectomy (RARP), it represents one task and not an entire procedure [9, 10]. Collectively, there is a need for clinically relevant objective metrics which can quantify a surgeon’s performance, provide feedback and ultimately improve both surgical and patient outcomes.

The idea of objective, clinically relevant metrics emerges with Clinically Relevant Performance Metrics (CRPMs) or Clinically Relevant Outcome Measures (CROMs) which have been explored to a limited degree in literature. CRPMs are applicable to a clinical context and can potentially correlate with patient outcomes. Specifically, they can inform trainee progression in the proctored operating phase of robotic training beyond simulation. In this review, we aimed to define CRPMs and assess their validity in robotic surgery training. As a secondary outcome, we aimed to compare the utility between CRPMs and existing measures of robotic performance, such as GEARS.

## Methods

This review was registered in May 2022 (PROSPERO ID: CRD42022332901). A systematic search of Medline and Embase databases was conducted in May 2022 following the PRISMA guidlienes. The search terms used were Clinically Relevant Performance Metrics (CRPMs) OR Clinically Relevant Outcome Measures (CROMs) AND robotic surgery. Additional articles were obtained via citation searching of included publications. After the exclusion of duplicate articles, two independent reviewers (MY, GT) initially screened articles based on title and abstract. Selection was completed by screening full-text articles based on eligibility criteria. Conflicts were resolved by a senior third independent reviewer (KL).

### Inclusion and exclusion criteria

The studies that were included addressed clinically relevant metrics including CRPMs, CROMs and clinically relevant metrics assessing intra-operative robotic performance. Studies assessing solely automated performance metrics (APMs), cognitive performance metrics (CPMs), patient-reported metrics or generalised measures of performance such as RACE, and GEARS were excluded. All settings of soft-tissue robot-assisted surgeries were included with dry laboratory, wet laboratory, animal models, and in-vivo operating. Articles addressing open surgery, laparoscopic surgery or not utilising a soft-tissue robot were excluded. Included studies investigated participants from multiple categories: surgeons (novice, experts), trainees (i.e. residents, interns), and medical students. Commentaries, conference abstracts, and reviews were excluded.

### Data extraction

For the included articles, data were extracted including, authors, study objective, context (speciality and operation), study design (participants and robotic setting), metric details, measurement of metrics, metric validation status, and comparison outcome data to existing methods of assessment (RACE and GEARS).

### Risk of assessment bias

A modified Newcastle–Ottawa scale was performed to assess the quality of included studies in this review (Appendix Table 3).

## Results

**Fig. 1:**
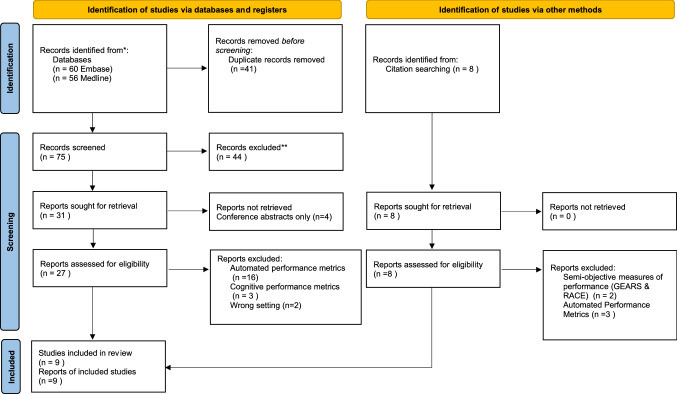
PRISMA diagram of the systematic search strategy

The initial database search yielded 116 articles with 75 unique articles remaining after the removal of duplicates. A further eight articles were retrieved through citation searching. After initial and full-text screening against eligibility criteria, nine studies were included in this review. Reasons for exclusion were the sole use of APMs, CPMs, subjective measures of performance, and utilising non-soft tissue robotics (see Fig. 1).

Individual study characteristics are summarised in Table 1. Included studies covered the specialities of urology (*n* = 5), coloproctology (*n* = 2), gastroenterology (*n* = 1) and the basic skills of robotic suturing and knot-tying (*n* = 1). Publication dates spanned the years 2017 to 2022. Together, their description of metrics included CRPMs, CROMs, PBP metrics, and reference-procedure metrics. Countries of publication included Germany [11, 12], England [13–17], and the USA [6, 18].

**Table 1:** Study characteristics

Authors journal and year	Specialty	Operation	Aims	Setting	No of participants	Metrics assessed
Witthaus et al. BJU Int (2020) [17]	Urology	NS-RARP	To incorporate and validate CRPMs into a hydrogel model for nerve-sparing robot-assisted radical prostatectomy	3D printing and PVA hydrogel NS-RARP simulation model	5 Experts (caseload > 500) 9 Novices (caseload < 50)	CRPMs Applied force to neurovascular bundle during dissection, Post-simulation margin status, UVA integrity, Estimated blood loss and Specific operating tasks (Bladder neck dissection, seminal vesicle dissection, nerve-sparing, bladder anastomosis)
Mottrie et al. BJU Int (2021) [14]	Urology	RARP with the classical anterior transperitoneal approach	To develop and seek consensus from procedure experts on the metrics that best characterise a RARP and determine if the metrics had response process evidence	Live surgery	*Study 1* Modified Delphi panel *n* = 19 *Study 2* 12 Experts (caseload > 500) 12 Novices (caseload < 10)	For a reference RARP, PBP metrics: 12 phases of the procedure; 81 steps; 245 procedural errors; 110 critical errors
Ma et al. J Urol (2022) [18]	Urology	UVA anastomosis	To test the feasibility of providing tailored feedback based upon CRPMs and explore its impact on the acquisition of robotic suturing skills	4 Dry lab UVA training sessions with and without feedback	Feedback group (*n* = 11) Control group (*n* = 12)	CRPMs: 4 APMs related to UVA step: master clutch usage, head out of console, camera move counts, and wrist articulation 4 suturing technical skill domains from RACE: needle positioning, needle entry, needle driving, and tissue approximation UVA integrity
Ghazi et al. BJU Int (2021) [15]	Urology	RAPN	To conduct a multi-institutional validation of a high-fidelity, perfused, inanimate, simulation platform for robot-assisted partial nephrectomy RAPN using CROMs	3D printing and PVA hydrogel RAPN simulation model	16 Experts (caseload > 150) 27 Novices (caseload < 30)	CROMs Total console time; Warm ischemia time; Estimated blood loss; Post-surgical margin status
Hussein et al. J Urol (2017) [6]	Urology	RARP	To develop and validate PACE to assess the quality of RARP	Live surgery	*Study 1* Modified Delphi panel *n* = 12 *Study 2* 28 Experts (attending surgeons) 28 Novices (chief residents, fellows)	Prostatectomy Assessment and Competence Evaluation (PACE) using 7 domains: (1) Bladder drop; (2)Prostate preparation; (3)bladder neck dissection; (4)posterior/seminal vesicle dissection; (5)neurovascular bundle preservation; (6)apical dissection;(7)ureterovesical anastomosis
Gómez et al. BJS Open (2022) [13]	Coloproctology	RA-LAR	To evaluate the use of binary PBP performance assessments and GEARS of RA-LAR procedure	Live surgery	7 Experts (caseload > 30) 5 Novices (caseload < 30)	For a reference RA-LAR PBP binary metrics: 14 procedure phases; 129 steps; 88 errors and 115 critical errors in women; 87 errors and 116 critical errors in men
Tou et al. Colorectal Dis (2020) [16]	Coloproctology	RA-LAR	To develop and operationally define performance metrics characterizing a reference approach RA-LAR	Delphi panel	Modified Delphi panel *n* = 18	For a reference RA-LAR PBP metrics: 14 procedure phases; 129 steps; 88 errors and 115 critical errors in women; 87 errors and 116 critical errors in men
Puliatti et al. Surg Endo (2021) [12]	General Robotic Surgery	Robotic suturing and knot tying anastomoses	To develop objective performance metrics for basic surgical skills training in robotic surgery	Wet lab—chicken	*Study 1* Modified Delphi panel *n* = 13 *Study 2* 10 Urology Experts (caseload > 300) 9 Urology Novices (caseload < 5)	Reference approach to the suturing and knot tying in anastomotic models: 5 steps (posterior, left, right, anterior walls, and knotting);12 suturing operative errors; 5 knotting operative errors; Fail to progress; 4 critical errors (anastomosis leakage, needle/suture breakage, catheter fixation during anastomosis, task completion time within 40 min)
Schmidt et al. Surg Endo (2022) [11]	Gastroenterology	Enterotomy intestinal anastomoses	To develop a reliable OSATS score for linear-stapled, hand-sewn closure of enterotomy intestinal anastomoses (A-OSATS)	Wet lab—porcine	*Study 1* Modified Delphi panel *n* = 19 *Study 2* 8 Experts (OSATS GRS > 28; > 10 caseload) 24 Intermediates (OSATS GRS 19–27; caseload 1–10) 8 Novices (< 18; caseload 0)	Anastomoses—objective structured assessment of technical skills (A-OSATS), weighted and unweighted, PBP metrics 4 key steps (intestinal placement, creation of enterotomies, stapling, and closure of enterotomy); 15 sub steps identified

### Definition of clinically relevant performance metrics

Throughout the articles, there was a lack of a clear consensus or homogenous definition for clinically relevant performance metrics in robotic surgery. As a result, this explicit terminology was utilised in only three of the included papers. Witthaus et al., introduced CRPMs as “concepts to design a conceptual framework for incorporating measures pertinent to a surgical task within a high-fidelity procedural simulation construct” [17]. Ghazi et al., defined CROMs as measures that “extend beyond basic robotic skills training into procedure-specific training” and provide tailored feedback to allow surgeons to progress based on individualised capabilities [15]. Ma et al., stated that CRPMs were those utilized to provide procedure-tailored feedback for surgical training and therefore “expedite the acquisition of robotic suturing skills” for each individual surgeon [18]. Other terminology utilised in the included publications were “procedure specific assessment tools” that provided an objective assessment of robotic intraoperative performance and enabled tailored training feedback to achieve competency [6, 12]. A further 4 articles used the term proficiency-based progression (PBP) metrics [11, 13, 14, 16].

### Development of clinically relevant performance metrics

Individual details and the specific metrics assessed by each study are represented in Table 1. Witthaus et al., and Ghazi et al., took a similar approach in defining their metrics. They used hydrogel models in conjunction with the Da Vinci Surgical System to develop anatomically and mechanically validated simulation models [15, 17]. This enabled the incorporation of tailored clinically relevant performance metrics in training for nerve-sparing robot-assisted radical prostatectomy (NS-RARP) and Robot-assisted partial nephrectomy (RAPN). The metrics included: applied force to the neurovascular bundle during dissection, post-simulation margin status, UVA integrity, task-specific operating tasks, estimated blood loss [17] as well as console time, warm ischemia time (WIT), and positive surgical margins (PSMs) [15], respectively.

Methodology for developing clinically relevant metrics for UVA utilised pre-existing validated metrics including APMs and RACE score [18]. The remaining 6 articles used a modified Delphi process, to identify and describe specific metrics for a reference procedure. These reference procedures included RARP [6, 14], robot-assisted low anterior resection (RA-LAR) [13, 16], robotic suturing and knot tying anastomosis [12], and intestinal anastomosis [11]. To create the reference metrics, a modified Delphi methodology using a panel of experts, outlined a combination of domains, procedure phases, steps, errors and critical errors. The metrics were edited, and a level of consensus was established before the final metrics were voted upon and finalised [14]. This is the only example in the literature of a structured approach to the development of clinically relevant performance metrics.

### Validation of clinically relevant performance metrics

#### Content validation

Content validity is defined as “the degree to which elements of an assessment instrument are relevant to a representative of the targeted construct for a particular assessment purpose” [19]. For clinically relevant metrics, this refers to how accurately they reflect performance in the clinical context they were intended to measure. CRPMs for NS-RARP were content validated by performing nerve sensor calibration, surgical margin verification and using the standard 180 ml UVA leak test [17]. An iterative development process was used to assess feedback and the feasibility of the CROMs in relation to the RAPN [15]. APMs related to UVA steps were collated from data from the Da Vinci robotic system, and combined with technical skill scores from RACE, which was previously validated [18]. Considering the articles that utilised a Delphi panel to create their reference metrics, content validation was achieved by voting upon each metric, and ensuring high-level consensus was achieved before the metrics were accepted and included as part of the finalised reference metrics [6, 11–14, 16]. Content validation measures for each study is represented in Table 2.

**Table 2:** Validity of metrics

Authors journal and year	Reliability assessment	Content validation	Construct validation	Criterion validation
Witthaus et al. BJU Int (2020) [17]	x	Nerve sensor calibration, surgical margin verification, and standard UVA leak testing (180 ml)	Experts outperformed novices across all metrics except EBL	Positive correlation between RACE scores, GEARS scores, and CRPMs
Mottrie et al. BJU Int (2021) [14]	Video recording of procedures evaluated by two blinded raters using metrics (IRR > 0.8)	High-level consensus reached on final RARP metrics by Delphi panel	Experts (with fewest errors) outperformed novices and lower-half experienced surgeons (most errors) across all metrics except procedural steps	x
Ma et al. J Urol (2022) [18]	Suturing technical skill scores were evaluated independently by two blinded raters (IRR > 0.8)	APMs: derived data of the DaVinci R robotic system RACE scores previously validated Standard UVA leak testing (180 ml)	Feedback group outperformed the control group across training sessions in all metrics except the needle entry score	x
Ghazi et al. BJU Int (2021) [15]	x	An iterative development process of pilot testing and revision was used for feedback and feasibility of metrics	Experts outperformed novices across all CROMs except for positive surgical margins	Positive correlation coefficient between each CROMs and total GEARS score
Hussein et al. J Urol (2017) [6]	Video recording of procedures evaluated by three blinded raters using metrics (ICC > 0.4)	High-level consensus reached on PACE metrics by Delphi panel	The expert group outperformed the trainees in all domains but only reached statistical significance for bladder drop, preparation of prostate, seminal vesicle and posterior plan dissection, and NVB preservation	x
Gómez et al. BJS Open (2022) [13]	Video recording of procedures evaluated by two blinded raters using metrics (Mean IRR = 0.94)	High-level consensus reached on the final RA-LAR metrics by Delphi panel	Experts (with fewest errors) outperformed novices and experienced surgeons (with most errors) across all reference metrics except procedural steps	The binary metrics demonstrated improved IRR and discrimination between surgical skill than GEARS
Tou et al. Colorectal Dis (2020) [16]	IRR > 0.8 of Delphi panel	High-level consensus reached on the final RA-LAR metrics by Delphi panel	x	x
Puliatti et al. Surg Endo (2021) [12]	Video recording of procedures evaluated by two blinded raters using metrics (Mean IRR = 0.92)	High-level consensus reached on all metrics by Delphi panel	The expert group outperformed the trainees in all domains	x
Schmidt et al. Surg Endo (2022) [11]	Video recording of procedures evaluated by two blinded raters using weighted (ICC = 0.923) and unweighted metrics (ICC = 0.924)	High-level consensus reached on all metrics by Delphi panel	Unweighted and weighted A-OSATS could differentiate between levels of surgical experience when categorised by OSATS GRS	x

*ICC* Intra-class correlation, *IRR* Inter-rater reliability

#### Construct validation (response process evidence)

Construct validation refers to the ability of CRPMs to differentiate between surgical skill, such as novices, intermediates and experts. All studies demonstrated that their metrics were able to distinguish between skill levels, though not all reached statistical significance (see Table 2).

Witthaus et al. showed that experts outperformed novices on all NS-RARP CRPMs including reduced nerve forces applied and total energy, superior margin results (*p* = 0.011), UVA integrity and all task-specific operating times except seminal vesicle dissection. Although not statistically significant, experts had a reduced EBL [17]. Similarly, Ghazi and colleagues demonstrated construct validity of their RAPN CROMs whereby experts significantly outperformed novices in all metrics, except for positive surgical margins [15]. Ma et al. found the feedback group, which received tailored feedback based on the CRPMs from UVA training tasks, outperformed the control group across all metrics except the needle entry score [18]. In addition to this, the effect size was measured to detect which metrics were more sensitive in detecting differences between the control and feedback group. For the UVA task, needle positioning, tissue approximation, and master clutch usage were found to have a higher effect size [18]. PACE was also found to have construct validity for RARP with the expert group outperforming the novices across all seven domains [6]. Puliatti et al. demonstrated construct validity for the reference approach to suturing and knot tying in anastomotic models, where novices had an increased mean task completion time, mean number of errors, and anastomotic leakage in comparison to experts [12]. Novices were also 12.5 times more likely to fail to progress throughout the task [12].

All the above studies used a caseload of procedures to differentiate between novice, intermediate and expert surgeons. Mottrie et al. and Gómez et al., however, found that within their expert surgeon groups, there existed two distinct populations: experienced surgeons with few errors and experienced surgeons with high errors [13, 14]. Those with the most errors demonstrated considerable performance variability, some performing worse than the weakest performing novice [13, 14]. To account for this variability, both studies considered two distinct populations. They found that experienced surgeons with the fewest errors performed significantly better across the metrics than those with high errors and novices, confirming construct validity [13, 14]. The neurovascular bundle dissection phase of the RARP and the rectal dissection in RA-LAR discriminated best between the total experienced surgeons and novices [13, 14]. Lastly, Schmidt et al. found that both the weighted and unweighted forms of the A-OSATS metric were unable to distinguish between surgical skill level according to caseload alone but achieved construct validity when participants were assigned to each skill level according to the OSATS global rating score (GRS) [11].

#### Criterion validity

Criterion validity refers to the relationship of CRPMs with other variables such as the validated semi-objective scoring systems, GEARS and RACE. Three studies examined the criterion validity of their metrics (Table 2). Witthaus et al. found that reduced force to neurovascular bundle during dissection correlated to higher force sensitivity (*p* = 0.019)) and total GEARS score (*p* = 0.000) [17]. UVA leak rate was also found to correlate with the total RACE score (*p* = 0.000) [17]. Ghazi and colleagues also found similar correlations between their CROMs and total GEARS score including console time, WIT, EBL and PSMs [15]. Gómez et al. found that GEARS had poor inter-rater reliability (IRR) for video scoring and weaker discrimination between surgical skill groups [13]. They concluded that PBP binary metrics demonstrated superior IRR than GEARS and robust discrimination amongst skill level, especially for total errors [13].

### Clinical context

Schmidt et al. constructed weighted A-OSATS scores which highlighted steps pertinent for patient outcomes but did not explore its predictive capabilities in comparison to the unweighted score [11]. Collectively, no study investigated the correlation between clinically relevant performance metrics and patient outcomes, though was highlighted as a point for future research.

## Discussion

Whilst the use of robotic surgery is increasing in clinical practice, training in robotic surgery and robotic skill assessments continue to require fundamental standardisation [20, 21]. For efficiency purposes, standardised robotic skill assessments should be readily available, operation-specific, objective and reproducible [20]. Having standardised and validated metrics is crucial for the development of safe proficiency-based robotic surgery training curricula [5]. In 2015, the first validated robotic training curriculum was developed which outlined training steps beginning with a baseline evaluation, simulation training, and observation of live operations [22]. This curriculum has not been tailored to specific operative procedures, and limitations include the inability to be objectively assessed, benchmarked and the lack of metrics for quality assurance [5]. Currently, metrics have been developed, such as automated performance metrics or semi-objective tools such as GEARS, that do provide overall robotic technical proficiency feedback, albeit lack transition to a clinical context. To investigate this current deficiency in standardised performance metrics, this review presents the findings of clinically relevant performance metrics with promising validity and the ability to provide tailored feedback.

It has become apparent that CRPMs lack a clear definition. Throughout this review, an emerging pattern of terminology associated with CRPMs or CROMs has emerged including objective assessment, proficiency-based progression, context-specific performance, competency training and tailored intra-operative feedback. Hence, we suggest that CRPMs can be defined as “context-specific metrics that objectively assess proficiency in robotic surgery training and provide tailored surgical feedback”.

Standardisation of robotic surgery training with objective performance metrics will allow easier detection of sub-optimised technique. This could translate to earlier post-operative complication detection and improved patient outcomes [5, 23, 24]. Given the heterogeneous development of CRPMs, it is important to identify which method is the most efficient and objective whilst still maintaining validity. Metrics that were identified in the review can be classified and divided into two groups: those that were procedure-specific or those that are generalisable to any operative procedure. Metrics identified as generalisable included applied force, post-simulation margin status, estimated blood loss, APMs, total console time/task completion time, warm ischemia time, and needle/suture breakage which constituted the CRPMs described by three studies [15, 17, 18]. It is not yet clear how performance differs with general versus specific procedure-based metric feedback. Given the aim of proficiency-based training it would be ideal to incorporate these clinically relevant metrics into a standard procedural description that can objectively assess both general and procedure-specific skills.

Proficiency based performance (PBP) metrics are defined as “objective and validated performance metrics to track progression of the trainee and operative skill on a specific task or procedure” and “allows learners to progress in their training based on their proficiency, rather than the number of cases performed or duration of practice” [13, 14, 16]. Four of the studies presented in this review used “PBP metrics” with enabled the development of reference metrics covering all domains of a surgical procedure and were found to have content and construct validity [11, 13, 14, 16]. An important element of PBP is sustained deliberate practice (SDP) which is the process of continuous training and repetition of robotic surgical skills that are both defined and assessed by PBP metrics [5, 25]. SDP has been shown to reduce error rates by 50% during robotic surgery training [25]. However, SDP requires the skills to be outlined by CRPMs that are agreed upon by the trainer and trainee in order for skill learning to be efficient [26]. From the studies presented, it appears the optimal way to ensure consensus and content validation of metrics is by using a modified Delphi methodology for procedure deconstruction, development of a standardised procedural description and identification of specific procedural phases, steps, and critical errors. Once reference PBP metrics have been produced via Delphi methodology, the development of simulation models that reflect the metrics can be created. As a result, SDP can be established through the continuum of proficiency-based training [5]. This is highlighted by Puliatti and Schmidt et al., using animal simulation models reflecting their suturing and knot tying reference metrics and A-OSATS metrics, respectively [11, 12].

Robotic surgery simulation using 3D models enables higher reproducibility of relevant anatomy and physiology of specific operative procedures in comparison to other models [5]. These 3D models enable the incorporation of CRPMs, a chance for improved SDP and proficiency-based training, as well as a smoother transition from simulation to a live-operating context [5]. Novel 3D simulation models are cost-effective as they do not need wet-lab facilities and are also more accessible for training in comparison to attending live surgeries. These 3D models can support SDP across various settings and enable real-time feedback that can be tailored to trainee performance [5]. Both Witthaus et al. and Ghazi et al. used 3D PVA hydrogel models to reflect NS-RARP and RAPN procedures, respectively. However, the CRPMs they incorporated were more generalised and could benefit by introducing PBP reference metrics deconstructing the crucial steps, and errors of each operation using a Delphi methodology [15, 17]. Promoting robotic surgery simulation training and preventing trainees that are early on their learning curve being exposed to patient surgeries, can result in a “reduction of surgical errors leading to an overall decrease in prolonged surgeries, and serious patient injury or death”, as defined by the ECRI institute [27]. Collectively, from the current data presented, using the Delphi methodology to develop CRPMs to aid in proficiency-based progression and incorporating CRPMs into novel full-immersion simulation using 3D printed models, represents the most standardised process of assessing proficiency in robotic surgery training. The CRPMs can then be translated for use in clinical contexts, standardising surgical assessment from simulation to live operations. In turn, this provides a structured methodology for developing future robotic surgery training curricula, tailored for different operative contexts.

The secondary aim of this review was to compare the utility of CRPMs to existing measures of performance, such as the semi-objective GEARS tool. It has been found that despite its ready use in robotic surgery training, low IRR for GEARS assessment has begun to appear in literature [13, 28]. In this review, it was highlighted that GEARS had poor inter-rater reliability for video scoring and weaker discrimination between surgical skill groups in comparison to PBP binary metrics which demonstrated good IRR and robust discrimination amongst skill level. This supports the view that PBP metrics may represent a more efficient, and objective tool than GEARS in assessing surgical skill throughout robotic surgical training. Supporting these findings, Satava and colleagues found that binary PBP metrics were superior in assessing “quality of assessment” in comparison to using a Likert scale such as GEARS for robotic surgery training of basic skills [29]. However, due to the lack of a “gold standard” robotic surgery training method, it is necessary to evaluate novel CRPMS in relation to existing measures of performance that are being developed currently, not exclusively GEARS. A cross-method validity may be a viable option to infer the relative utility of novel robotic surgery metrics [30]. For example, a study by Hung and colleagues found a strong correlation between APMs and GEARS during RARP though stressed that a lack of statistical correlation between the two did not suggest superiority of either metric [31]. They suggested that refined clinical metrics correlated to clinical outcomes could help delineate superiority [31].

## Limitations

This review aimed to evaluate the current use of CRPMs for robotic surgery training. A possible limitation is the utilisation of a single mode of metric evaluation narrows the available scope of feedback for trainees. Other forms of performance metrics exist including cognitive performance metrics, eye-tracking metrics and even APMs, that were not explored in this review. Ideally, all these metrics can be evaluated on their use in conjunction with one another, to determine if a synergistic effect exists in optimising trainee performance and translation to a clinical context. Future studies can explore a multimodal metric evaluation in simulation as well as in-vivo training in robotic surgery and its association with progression trainee performance.

Despite exploring CRPMs in this review, they have not been translated to a clinical context as they were indented. Patient outcome data has, however, been explored by Hung and colleagues in relation to APM’s and their correlation with early urinary continence after RARP [32]. They found that whilst clinical factors confounded patient outcome data, specific surgeon kinematic metrics including velocity and wrist articulation served as independent predicators of urinary continence after RARP. However, this research came after the extensive development and validation of APM’s for RARP [33]. Likewise, studies in this review are in the early stages of optimising their CRPMs and hope to explore the relation of their metrics to patient outcomes in a future study. In general, it has been found that skill level, rather than caseload, is a better predictor of both intra-operative performance and clinical outcome [13, 34, 35]. Therefore, future studies exploring construct-validated CRPMs and their association with clinical outcomes is promising.

Finally, the studies in this review were limited by small sample sizes and reduced power. The modified NOS scale for non-randomised studies identified two good-quality studies [15, 18], with the remaining seven being of poor quality. Most studies in this review were prospective cohort studies except for one unblinded randomised control trial by Ma et al. [18]. Future studies incorporating the validated CRPMS presented here will benefit from larger sample sizes to detect power and randomised controlled trials to build high-quality validity evidence for this approach.

## Conclusion

This study highlights the described clinically relevant performance metrics in the setting of robotic surgery. There is significant scope for the development and validation of clinically relevant metrics in this context. Clinically relevant performance metrics can assist in the development of proficiency-based progression curricula that can be carried across from a simulation setting into clinical practice.

## Data Availability

The material in the manuscript is the original work of the authors and has not been presented or submitted elsewhere for publication.

## Appendix Table 3: Risk of assessment bias using the modified NOS scale

See Table 3.

**Table 3:** Modified Newcastle—Ottawa quality assessment scale cohort studies

No.	Criterion	Decision rule	Score (* = 1, no* = 0)								
Selection			Witthaus et al. (2019)	Motrrie et al. (2020)	Ma et al. (2022)	Ghazi et al. (2020)	Hussein et al. [6]	Gómez et al. [13]	Tou et al. [16]	Puliatti et al. [12]	Schmidt et al. [11]
1	Representativeness of the exposed cohort	(a) Consecutive eligible participants were selected, participants were randomly selected, or all participants were invited to participate from the source population* (b) Not satisfying requirements in part (a), or not stated	b	b	a*	a*	b	a*	a*	a*	a*
2	Selection of the non-exposed cohort	(a) Selected from the same source population* (b) Selected from a different source population (c) No description	a*	a*	a*	a*	a*	a*	a*	a*	a*
3	Ascertainment of exposure	(a) Structured injury data (e.g. record completed by medical staff)* (b) Structured interview* (c) Written self-report (d) No description	a* (video)	a* (video)	A*(Feedback through interview)	a*	a* (video)	a* (video)	N/A	a* (video)	a* (video)
4	Demonstration that the outcome of interest was not present at the start of the study	(a) Yes* (b) No or not explicitly stated	b	b	b	b	b	b	N/A	b	b
*Comparability*											
1	Comparability of cohorts on the basis of the design or analysis	(a) Study controls for previous injury* (b) Study controls for age* *Note:* Exposed and non-exposed individuals must be matched in the design and/or confounders must be adjusted for in the analysis. Alone statements of no differences between groups or that differences were not statistically significant are not sufficient	a* (caseload)	a* (caseload)	a* (caseload, surgical training) b* (age)	a* (caseload, sex, position)	Nil	Nil	N/A	a* (caseload) b* (age)	a* (surgical skill level, caseload) b* (age)
*Outcome*											
1	Assessment of outcome	(a) Independent or blind assessment stated, or confirmation of the outcome by reference to secure records (e.g. imaging, structured injury data, etc.)* (b) Record linkage (e.g. identified through ICD codes on database records)* (c) Self-report with no reference to original structured injury data or imaging (d) No description	a*	a*	a*	a*	a*	a*	N/A	a*	a*
2	Was follow-up long enough for outcomes to occur?	(a) Yes (≥ 3 months)* (b) No (< 3 months)	b	b	a*	b	b	b	N/A	b	b
3	Adequacy of follow up of cohorts	(a) Complete follow up – all participants accounted for* (b) Subjects lost to follow up unlikely to introduce bias (< 15% lost to follow up, or description provided of those lost*) (c) Follow up rate < 85% and no description of those lost provided] (d) No statement	d	d	d	a*	d	d	N/A	d	d
		Score	4	4	7	6	3	4	2	6	6

Score (*=1, if no star zero score)
